# The link between diabetes and male infertility: mechanisms and implications

**DOI:** 10.1038/s41598-026-63095-w

**Published:** 2026-07-24

**Authors:** Hesham Haffez, Mohamed Mosaad, Ahmed Y. Rezk, Ahmed Sayed, Zeinab A. Hassan

**Affiliations:** 1 Biochemistry and Molecular Biology Department, Faculty of Pharmacy, Capital University (Formerly Helwan University), Cairo, 11795 Egypt; 2 Center of Scientific Excellence “Helwan Structural Biology Research, (HSBR)”, Capital University (Formerly Helwan) Helwan University, Cairo, 11795 Egypt; 3 Benha Fertility and ICSI Centre, Qalyubia, 13511 Egypt; 4 Obstetrics & Gynecology Department, Faculty of Medicine, Benha University, Qalyubia, 13511 Egypt; 5 Genomics Research Program, Children’s Cancer Hospital 57357, 11562 Cairo, Egypt; 6 Capital University (Formerly Helwan University), Cairo, 11795 Egypt

**Keywords:** Diabetes mellitus, Male infertility, Apoptosis, Semen, Hormones, Diseases, Endocrinology, Medical research

## Abstract

Diabetes mellitus (DM) is a growing global health crisis affecting male reproductive function, yet the enhanced combined effects of diabetes with pre-existing fertility abnormalities remain poorly understood. This study aims to investigate the individual and combined effects of diabetes and abnormal semen parameters on male reproductive function and the underlying apoptotic mechanisms. Thirty-nine men were enrolled and stratified into two phases: a validation cohort (nondiabetic vs. diabetic; fertile vs. abnormally infertile) and a four-group mechanistic study (normal nondiabetic (*n* = 10), abnormal nondiabetic (*n* = 11), normal diabetic (*n* = 10), and abnormal diabetic (*n* = 8)). We assessed HbA1c, FSH, LH, testosterone, semen parameters, sperm apoptosis (Annexin V/PI flow cytometry), DNA fragmentation (diphenylamine), and expression of apoptotic markers (caspase-3, cytochrome c, Bax, and Bcl-2) at gene and protein levels. Diabetic patients had significantly higher HbA1c (9.6 ± 2.31% vs. 5.49 ± 0.42%, *p* < 0.001), elevated FSH and LH, and reduced testosterone. Abnormal infertile patients showed impaired motility and morphology. The abnormal diabetic group had the most severe dysfunction: lowest rapid progressive motility (11.3 ± 3.10%, *p* < 0.001 vs. control) and highest early apoptosis (58.9 ± 8.79%, *p* < 0.0001). Protein analysis showed a pro-apoptotic shift with an elevated Bax/Bcl-2 ratio (3.64), increased cytochrome-c, and caspase-3 activation exclusively in this group (*p* < 0.0001). Diabetes and abnormal semen parameters exert enhanced combined detrimental effects on male fertility via enhanced activation of the intrinsic mitochondrial apoptotic pathway. Diabetic men with pre-existing fertility abnormalities exhibit the most severe phenotype and require prioritized clinical intervention.

**Supplementary Information:**

The online version contains supplementary material available at 10.1038/s41598-026-63095-w.

## Introduction

Diabetes mellitus (DM) represents one of the most pressing public health challenges of the twenty-first century, with its prevalence reaching epidemic proportions worldwide. According to the International Diabetes Federation, approximately 537 million adults aged 20–79 years were living with diabetes in 2021, and this figure is projected to escalate to 643 million by 2030 and 783 million by 2045^1^. The most recent data indicates that the diabetes prevalence among the population aged 20 to 79 in Egypt was 22.4% in 2024^2^. This figure represents the percentage of adults with either type 1 or type 2 diabetes, with over 18% of Egyptian adults currently living with diabetes. Furthermore, the number of affected adults is projected to reach 20 million by 2045, highlighting a growing health crisis^2^. The burden is particularly acute in low- and middle-income countries, where healthcare infrastructure often struggles to accommodate the escalating demand for diabetes care. Concurrently, male infertility has emerged as a significant global health concern, affecting approximately 8–12% of couples of reproductive age worldwide, with male factors contributing solely to 30–50% of infertility cases and jointly to up to 30% of cases^3^. The World Health Organization recognizes infertility as a disease of the reproductive system, and its psychological, social, and economic ramifications extend far beyond the inability to conceive, affecting quality of life, marital stability, and mental health^4^. Notably, these two conditions are not mutually exclusive. Epidemiological evidence increasingly demonstrates that diabetic men exhibit significantly higher rates of subfertility and reproductive dysfunction compared to their non-diabetic counterparts^5^. This intersection is particularly concerning given the rising prevalence of both conditions globally and the tendency for diabetes to affect men during their peak reproductive years. Understanding the relationship between diabetes and male infertility has therefore become an urgent public health priority. This anticipated correlation is driven by multiple factors, including rapid urbanization, sedentary lifestyles, rising obesity rates, nutritional transitions toward Westernized dietary patterns, and an aging population^6,7^. The socioeconomic implications are profound, with diabetes imposing substantial direct healthcare costs and indirect productivity losses on individuals, families, and the national economy.

Clinical studies showed that diabetes adversely affects male reproductive function through multiple pathways^5,8,9^. Men with diabetes consistently demonstrate impaired semen parameters compared to non-diabetic controls, including reduced semen volume, diminished sperm concentration, decreased sperm motility, and increased morphological abnormalities. These alterations have been documented in both type 1 diabetes mellitus (T1DM) and type 2 diabetes mellitus (T2DM)^5,10,11^. Endocrine disturbances represent another well-recognised dimension of diabetes-associated reproductive dysfunction characterised by low testosterone with inappropriately normal or low luteinising hormone (LH) and follicle-stimulating hormone (FSH) levels^12^. Despite these established associations, significant knowledge gaps persist. First, most mechanistic studies have been conducted in animal models, with limited direct validation in human populations^13^. The translational relevance of findings from streptozotocin-induced diabetic rodents to human diabetic patients, particularly those with long-standing disease and multiple comorbidities, remains uncertain. Second, the molecular pathways linking hyperglycemia to germ cell dysfunction are incompletely elucidated, and the relative contributions of apoptotic signaling continue to be debated. Third, and perhaps most critically, the enhanced combined effects of diabetes with pre-existing fertility abnormalities have received insufficient attention. In clinical practice, diabetic men presenting for fertility evaluation represent a heterogeneous population: some have normal baseline fertility with acquired diabetes-related impairment, while others have pre-existing reproductive abnormalities that may be exacerbated by diabetes. Understanding how these conditions interact, whether additively or synergistically, is essential for accurate prognosis, patient counselling, and treatment planning.

Apoptosis is among the various mechanisms proposed to explain diabetes-associated male infertility as a particularly compelling candidate^14^. The intrinsic (mitochondrial) apoptotic pathway appears especially relevant in the context of diabetes. This pathway is regulated by the Bcl-2 family of proteins, which includes both anti-apoptotic members such as Bcl-2 and pro-apoptotic members such as Bax^15,16^. The balance between these opposing forces, often expressed as the Bax/Bcl-2 ratio, determines mitochondrial outer membrane permeability, cytochrome c release, and subsequent activation of the caspase cascade leading to cell death^17,18^. Experimental evidence from animal models suggests that diabetes shifts this balance toward apoptosis, with increased Bax expression, decreased Bcl-2 expression, elevated Bax/Bcl-2 ratios, and enhanced caspase-3 activation in testicular tissue^19^. Whether similar apoptotic dysregulation occurs in human diabetic men, and whether it is exacerbated by co-existing fertility abnormalities, remains to be definitively established. Elucidating these relationships could identify novel therapeutic targets and inform the development of interventions to preserve fertility in diabetic populations.

The clinical implications of diabetes-associated male infertility extend beyond individual patient care. Sperm quality has emerged as a potential biomarker for overall male health, with meta-analyses demonstrating that men with higher sperm counts and better morphology have significantly lower risks of cardiovascular disease, T2DM, and all-cause early mortality^20^. This association suggests that reproductive dysfunction may serve as an early indicator of systemic metabolic compromise, offering opportunities for preventive intervention.

Therefore, the present study is designed to address these knowledge gaps by comprehensively investigating the individual and combined effects of diabetes and abnormal semen parameters on male reproductive function. Building upon initial patient validation, participants will be classified into four distinct groups: normal nondiabetic, abnormal nondiabetic, normal diabetic, and abnormal diabetic. This design enables dissection of the independent contributions of diabetes and pre-existing infertility, as well as their potential enhanced combined interactions. The combination of diabetes and abnormal semen parameters would produce exacerbated reproductive dysfunction through enhanced activation of the intrinsic apoptotic pathway. To test this hypothesis, the study will evaluate: (1) conventional semen parameters and hormonal profiles across groups; (2) sperm apoptosis via flow cytometry and DNA fragmentation; (3) gene expression of inflammatory and apoptotic mediators (IL-6, Caspase-3, Cytochrome c, Bax, and Bcl-2); and (4) corresponding protein expression levels. By integrating clinical, cellular, and molecular analyses, this study aims to provide mechanistic insights into diabetes-associated male infertility and identify potential therapeutic targets for this increasingly prevalent condition.

## Results

### Patient validation and baseline characteristics

**Fig. 1:**
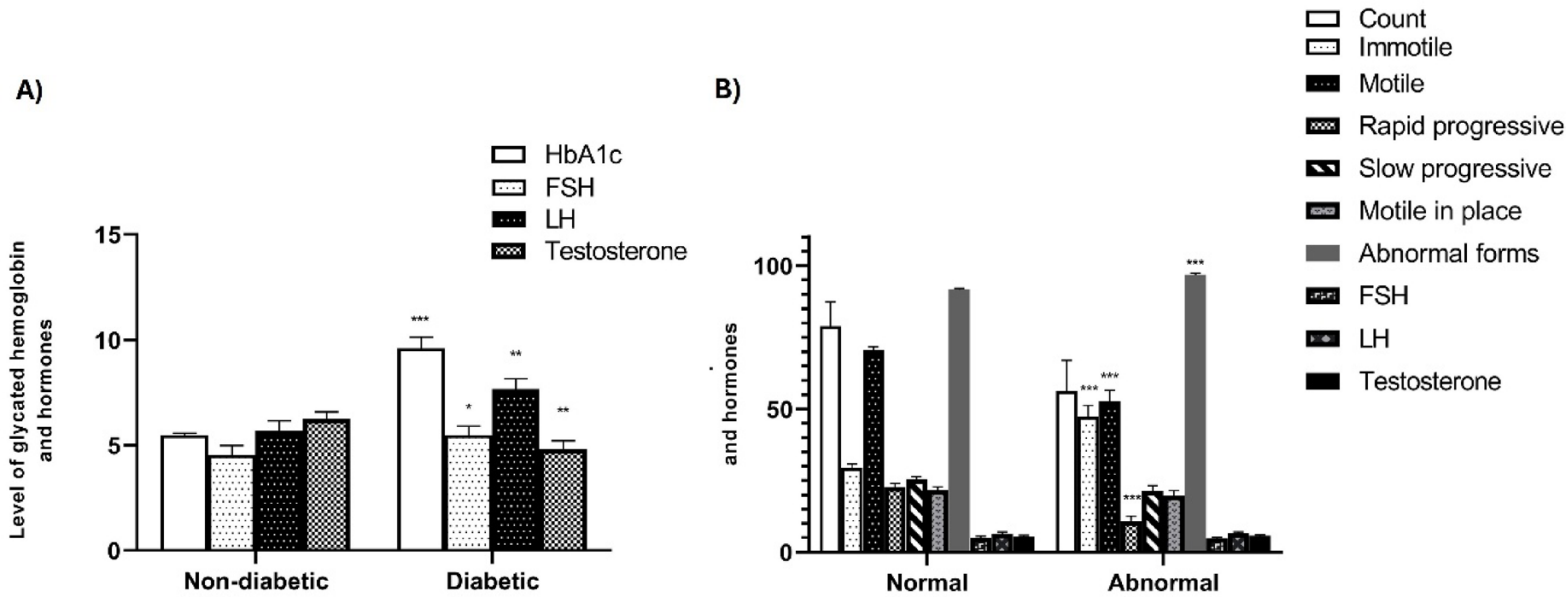
Representation of (**A**) Glycated hemoglobin (%HbA1c), hormonal and (**B**) Semen profiles of patients classified as diabetic and abnormal infertile patients compared to healthy control. Diabetic patients showed significantly elevated glycated hemoglobin, FSH, LH levels with reduced total testosterone level. Abnormal infertile patients showed elevated percent of immotile and abnormal sperms with reduced percent of motile and rapidly progressive sperms. All experiments were performed in replicates, and statistical analysis was performed using un-paired t-test with post hoc tests. *P < 0.05, **P < 0.01, ***P < 0.001, ****P < 0.0001.

To evaluate the validity of the patient selection, the demographic data, glycemic, hormonal, and seminal parameters of the selected diabetic and abnormal, infertile patients were compared against their respective control groups. The data are summarized in Table 1; Fig. 1.

#### Validation of diabetic patient group

The diabetic patient group (*n* = 18) and the nondiabetic control group (*n* = 21) were comparable in age (38.28 ± 0.99 vs. 36.15 ± 1.44 years, *p* = 0.498). As expected for the validation of diabetes, the diabetic patients exhibited significantly poorer glycemic control, with a markedly elevated mean HbA1c level (9.6 ± 0.55%) compared to the nondiabetic controls (5.49 ± 0.09%, *p* < 0.001) (Table 1; Fig. 1A). Furthermore, the diabetic group displayed a distinct hormonal profile. They had significantly higher levels of FSH (5.499 ± 0.42 vs. 4.549 ± 0.44 mIU/mL, *p* < 0.05) and LH (7.666 ± 0.49 vs. 5.708 ± 0.45 mIU/mL, *p* < 0.01), associated with a significantly lower total testosterone level (4.806 ± 0.40 vs. 6.258 ± 0.33 ng/dL, *p* < 0.01) (Table 1; Fig. 1A). Notably, the mean values of BMI were higher in diabetic patients than in non-diabetic, but the difference was not statistically significant (30.72 ± 1.16 vs. 28.71 ± 0.55 kg/m², *p* = 0.228). These findings may validate the metabolic and endocrine alterations associated with diabetes in the studied group.

**Table 1:** Demographic distribution data for validation of selection of diabetic and abnormal infertile patients compared to healthy control.

Validation of diabetes mellitus			
Patient parameters	Nondiabetic patients (*n* = 21), Mean ± SEM	Diabetic patients (*n* = 18), Mean ± SEM	*P-value*
Age (years)	36.15 ± 1.44	38.28 ± 0.99	0.498
BMI (kg/m^2^)	28.71 ± 0.55	30.72 ± 1.16	0.228
HbA1c (%)	5.49 ± 0.09	9.6 ± 0.55***	< 0.001
FSH (mIU/mL)	4.549 ± 0.44	5.499 ± 0.42*	0.0329
LH (mIU/mL)	5.708 ± 0.45	7.666 ± 0.49**	0.0016
Total testosterone (ng/dL)	6.258 ± 0.33	4.806 ± 0.40**	0.0045
Validation of male abnormality			
Semen profile	Normal fertile patients (*n* = 20) Mean ± SEM	Abnormal infertile patients (*n* = 19) Mean ± SEM	*P-value*
Age (years)	37.85 ± 1.02	36.55 ± 1.56	0.31
BMI (kg/m^2^)	30.0 ± 0.60	29.4 ± 1.21	0.285
Count	79 ± 8.27 × 10^6^	56.2 ± 10.79 × 10^6^	0.1170
Immotile sperm	29.50 ± 1.30	47.37 ± 3.96***	0.0003
Motile sperm	70.50 ± 1.30	52.63 ± 3.96***	0.0003
Rapidly progressive	22.75 ± 1.28	10.79 ± 1.80****	< 0.0001
Slowly progressive	25.50 ± 0.95	21.32 ± 1.98	0.0992
Motile in place	21.75 ± 1.10	19.74 ± 1.85	0.4164
Abnormal form	91.60 ± 0.47	96.74 ± 0.566****	< 0.0001
Testosterone	5.464 ± 0.44	5.718 ± 0.35	0.4736
FSH	5.090 ± 0.50	4.879 ± 0.36	0.7387
LH	6.451 ± 0.66	6.781 ± 0.30	0.6552

The validation criteria include glycemic control (HbA1c), hormone profile (FSH, LH and total testosterone) and semen profile (count, immotile sperm, motile sperm, rapidly progressive, slowly progressive, motile in place, abnormal form). Diabetic patients showed significant elevation of % HbA1c level while, abnormal infertile patients showed elevated percent of immotile and abnormal sperms with reduced percent of motile and rapidly progressive sperms. All experiments were performed in replicates, and statistical analysis was performed using un-paired t-test.

*P < 0.05, **P < 0.01, ***P < 0.001, ****P < 0.0001.

#### Validation of abnormal infertile patient group

**Fig. 2:**
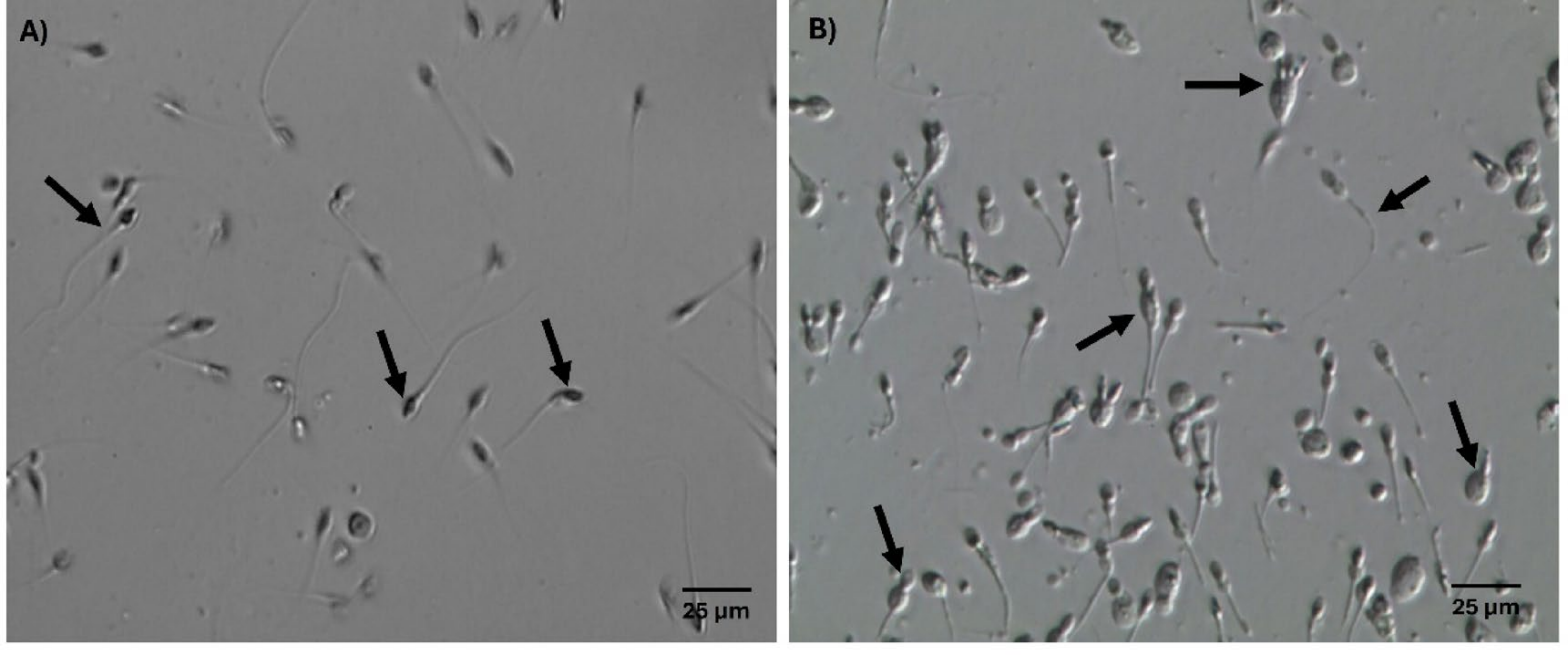
Representative micrographs of normal and abnormal human sperm morphology associated with male infertility. (**A**) Normal spermatozoa with an oval head, intact acrosome, well-defined midpiece, and a single long tail (arrows shown), and (**B**) Abnormal sperm morphology characterized by head defects (amorphous and enlarged heads) and cytoplasmic droplet retention midpieces (arrows shown). Images were taken as phase-contrast microscopy images of human spermatozoa by OCTAX EyeWare 2.2.2.318 (scale bar = 25 μm).

The abnormal infertile patient group (*n* = 19) and the normal fertile control group (*n* = 20) were well-matched for both age (36.55 ± 1.56 vs. 37.85 ± 1.02 years, *p* = 0.31) and BMI (29.4 ± 1.21 vs. 30.0 ± 0.60 kg/m², *p* = 0.285), (Table 1). The validation of their abnormal fertility status was confirmed by significant differences in multiple semen parameters. The abnormal infertile group demonstrated a significantly higher percentage of immotile sperm (47.37 ± 3.96% vs. 29.50 ± 1.30%, *p* < 0.001) and lower percentage of motile sperm (52.63 ± 3.96% vs. 70.50 ± 1.30%, *p* < 0.001) (Table 1; Fig. 1A). Specifically, the proportion of rapidly progressive sperm was significantly reduced in the abnormal infertile group (10.79 ± 1.80% vs. 22.75 ± 1.28%, *p* < 0.0001). Additionally, this group exhibited a significantly higher percentage of sperm with abnormal morphology (96.74 ± 0.57% vs. 91.60 ± 0.47%, *p* < 0.0001). Although sperm count was lower in the abnormal infertile group (56.24 ± 10.80 million vs. 79.00 ± 8.27 million), this difference did not reach statistical significance (*p = 0.117*) (Table 1; Fig. 1A). In contrast to the diabetic group, the hormonal profile (testosterone, FSH, and LH) did not differ significantly between the abnormal infertile and normal fertile groups. The phase contrast microscopic examination of semen samples revealed marked heterogeneity in sperm concentration and morphology across the analyzed fields (Fig. 2). Normal samples demonstrated predominantly normal spermatozoa with intact oval heads and well-defined flagella (Fig. 2A, supplementary video 1), whereas Fig. 2B-F showed reduced sperm density despite largely preserved morphology suggesting decrease in sperm count consistent with oligozoospermia (supplementary video 2). Markedly reduced sperm count with scattered spermatozoa with microcephalic heads (supplementary video 3). Significant morphological abnormalities were observed with cellular debris, round cells, and poorly distinguishable sperm structures, including amorphous and enlarged heads with irregular midpieces, suggesting impaired spermatogenesis, (supplementary video 4). Also, triple tail abnormalities were observed (supplementary video 5). Additionally, mixed defects including coiled and bent flagella with sperm aggregation are indicative of compromised motility (supplementary video 6). Collectively, these findings highlight variable degrees of sperm dysfunction, encompassing reduced count and structural abnormalities associated with male infertility and validating their classification as infertile subjects.

### Subgroup analysis of diabetic and infertility phenotypes

**Fig. 3:**
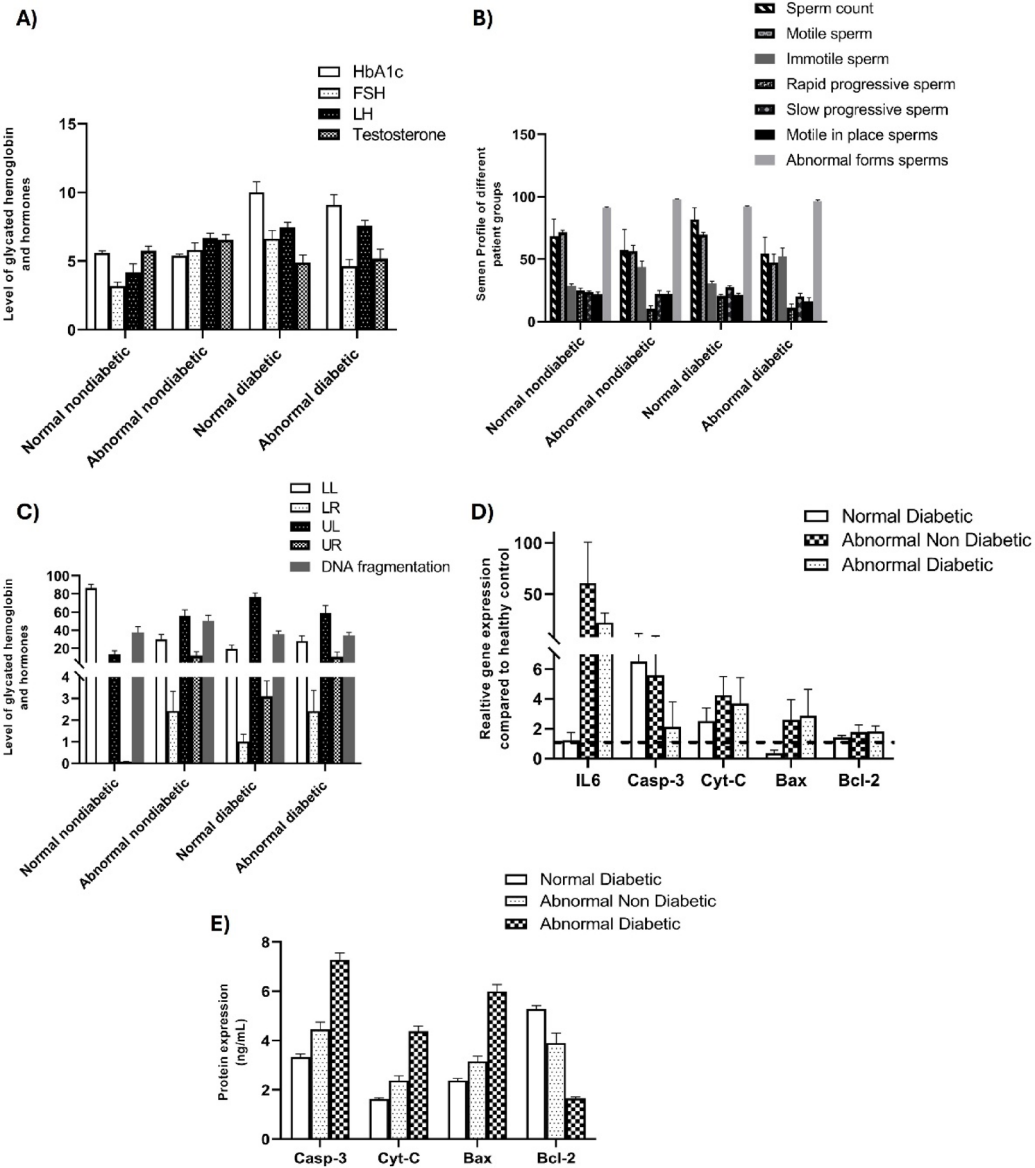
Effects of diabetes and semen quality on hormonal profile, sperm characteristics, DNA integrity, and apoptosis-related markers in four study groups: normal non-diabetic, abnormal non-diabetic, normal diabetic, and abnormal diabetic subjects. (**A**) Serum levels of glycated hemoglobin (HbA1c), follicle-stimulating hormone (FSH), luteinizing hormone (LH), and testosterone Diabetic groups show higher HbA1c levels and altered reproductive hormone profiles compared with non-diabetic controls, (**B**) Semen parameters including sperm count, total motile sperm, immotile sperm, rapid progressive motility, slow progressive motility, non-progressive motility (motile in place), and abnormal sperm morphology. Individuals with abnormal semen parameters, particularly diabetic subjects, show reduced sperm count and motility with increased abnormal forms, (**C**) Distribution of sperm apoptosis categories (LL, LR, UL, UR) and sperm DNA fragmentation. Abnormal and diabetic groups exhibit decreased progressive motility and increased DNA fragmentation compared with normal controls, (**D**) Relative mRNA expression of inflammatory and apoptosis-related genes (*IL-6*, *Casp-3*, *Cyt-C*, *Bax*, and *Bcl-2*) expressed relative to healthy controls, and (**E**) Corresponding protein expression levels (ng/mL) of Casp-3, Cyt-C, Bax, and Bcl-2. All data are replicated and presented as mean ± SEM. The quantitative statistical analysis was performed using one-way ANOVA with post hoc tests and are presented in Table 2.

Following the initial validation, patients were further stratified into four distinct groups to delineate the individual and combined effects of diabetes and abnormal semen parameters: normal nondiabetic (control, *n* = 10), abnormal nondiabetic (*n* = 11), normal diabetic (*n* = 10), and abnormal diabetic (*n* = 8). The demographic, clinical, and molecular profiles of these groups are presented in Table 2; Fig. 3.

#### Glycemic and hormonal profiles

As anticipated, both diabetic groups (normal diabetic and abnormal diabetic) exhibited significantly elevated HbA1c levels compared to their nondiabetic counterparts (*p < 0.0001* for all comparisons, Fig. 3A). The hormonal analysis revealed distinct patterns of reproductive hormone dysregulation. The abnormal nondiabetic group showed significantly higher FSH (5.8 ± 0.52 mIU/mL, *p* < 0.01) and LH (6.7 ± 0.36 mIU/mL, *p* < 0.01) compared to the normal nondiabetic group (FSH: 3.2 ± 0.3 mIU/mL; LH: 4.2 ± 0.61 mIU/mL). Interestingly, the normal diabetic group displayed an even more pronounced elevation in both FSH (6.6 ± 0.63 mIU/mL, *p* < 0.001) and LH (7.4 ± 0.39 mIU/mL, *p* < 0.0001). A significant difference in FSH was also observed between the two diabetic groups (normal diabetic vs. abnormal diabetic, *p* < 0.05). No significant differences in total testosterone levels were observed across any of the four groups.

#### Semen analysis

Analysis of semen parameters revealed the compounded impact of both conditions. While sperm count did not differ significantly across groups, motility and morphology were severely affected (Fig. 3B). The abnormal nondiabetic and abnormal diabetic groups both exhibited a significantly higher percentage of immotile sperm and a correspondingly lower percentage of total motile sperm compared to the normal nondiabetic group (*p < 0.05* and *p* < 0.01, respectively). The percentage of rapidly progressive sperm was markedly reduced in all abnormal groups. Notably, the abnormal diabetic group showed the most severe impairment, with a significant reduction compared not only to the normal nondiabetic control (*p < 0.001*) but also to the abnormal nondiabetic group (*p < 0.05*). Similarly, the percentage of sperm with abnormal morphology was significantly higher in all groups compared to the Normal nondiabetic control (*p < 0.0001*), with the abnormal nondiabetic group exhibiting the highest level (97.9 ± 0.34%).

**Table 2:** Demographic distribution data for patients classified into four groups (normal nondiabetic, nondiabetic, normal diabetic, and abnormal diabetic group).

Patient parameters	Normal nondiabetic group (*n* = 10), Mean ± SEM	Abnormal nondiabetic group (*n* = 11), Mean ± SEM	Normal diabetic group (*n* = 10), Mean ± SEM	Abnormal diabetic group (*n* = 8), Mean ± SEM	Significant only *P*-value
Age (years)	37.2 ± 1.59	35.1 ± 2.47	38.5 ± 1.25	38 ± 1.66	--
BMI (kg/m^2^)	29.7 ± 0.89	27.7 ± 0.44	30.3 ± 0.82	31.1 ± 2.37	--
HbA1c (%)	5.6 ± 0.14	5.4 ± 0.11	10 ± 0.78	9.1 ± 0.75	^2****^P < 0.0001, ^3***^P = 0.0002 ^4****^P < 0.0001, ^5****^P < 0.0001
FSH	3.2 ± 0.3	5.8 ± 0.52	6.6 ± 0.63	4.6 ± 0.48	^1**^P = 0.0042, ^2***^P = 0.0002 ^6*^P = 0.0350
LH	4.2 ± 0.61	6.7 ± 0.36	7.4 ± 0.39	7.6 ± 0.39	^1**^P = 0.0021, ^2****^P < 0.0001 ^3****^P < 0.0001
Total testosterone	5.8 ± 0.35	6.6 ± 0.37	4.9 ± 0.53	5.2 ± 0.67	--
Sperm count	68.5 ± 13.56 × 10^6^	57.5 ± 16.54 × 10^6^	81.9 ± 9.35 × 10^6^	54.5 ± 13.24 × 10^6^	--
Immotile sperm	28.5 ± 1.83	43.6 ± 4.91	30.5 ± 1.89	52.5 ± 6.47	^1*^P = 0.0387, ^3**^P = 0.0019 ^6**^P = 0.0041
Motile sperm	71.5 ± 1.83	56.4 ± 4.91	69.5 ± 1.89	47.5 ± 6.47	^1*^P = 0.0387, ^3**^P = 0.0019 ^6**^P = 0.0041
Rapidly progressive	25.0 ± 1.82	10.5 ± 2.28	20.5 ± 1.57	11.3 ± 3.10	^1***^P < 0.0001, ^3***^P = 0.0008 ^4**^P = 0.0074, ^6*^P = 0.0214
Slowly progressive	23.5 ± 1.1	22.3 ± 2.81	27.5 ± 1.34	20.0 ± 2.83	--
Motile in place	22.0 ± 1.86	22.3 ± 2.10	21.5 ± 1.30	16.3 ± 3.10	--
Abnormal form	91.2 ± 0.55	97.9 ± 0.34	92.0 ± 0.77	96.6 ± 0.98	^1****^P < 0.0001, ^3****^P < 0.0001 ^4****^P < 0.0001, ^6***^P = 0.0001
Apoptosis parameters					
LL parent (%)	86.4 ± 11.46	29.6 ± 16.69	19.5 ± 9.52	27.9 ± 15.71	^1****^P < 0.0001, ^2****^P < 0.0001 ^3****^P < 0.0001
UL parent (%)	13.5 ± 3.8	56.2 ± 6.49	76.4 ± 4.68	58.9 ± 8.79	^1****^P < 0.0001, ^2****^P < 0.0001 ^3****^P < 0.0001
UR parent (%)	0.07 ± 0.03	11.8 ± 4.6	3.12 ± 0.7	10.9 ± 5.17	--
LR parent (%)	0.01 ± 0.006	2.4 ± 0.91	1 ± 0.35	2.4 ± 0.98	--
DNA fragmentation					
DNA fragmentation (%)	37.8 ± 6.1	50.4 ± 6.4	35.6 ± 3.8	34.1 ± 3.6	--
Gene expression					
IL6	1.0	60.6 ± 40.11	1.230 ± 0.49	22.42 ± 9.13	^6*^P = 0.0158
Casp-3	1.0	5.58 ± 3.9	6.512 ± 5.2	2.13 ± 1.70	--
Cyt-C	1.0	4.26 ± 1.25	2.52 ± 0.87	3.69 ± 1.74	--
Bax	1.0	2.59 ± 1.36	0.36 ± 0.21	2.87 ± 1.78	--
Bcl-2	1.0	1.78 ± 0.47	1.41 ± 0.13	1.82 ± 0.37	--
Bax/Bcl-2	1.0	1.46	0.26	1.57	--
Protein expression (fold-change)					
Casp-3	5.7 ± 0.25	3.3 ± 0.13	4.5 ± 0.30	7.3 ± 0.28	^1****^P < 0.0001, ^2**^P = 0.0073 ^3**^P = 0.0027, ^4**^P = 0.0080 ^5****^P < 0.0001, ^6****^P < 0.0001
Cyt-C	3.45 ± 0.14	1.63 ± 0.04	2.38 ± 0.2	4.38 ± 0.21	^1****^P < 0.0001, ^2**^P = 0.0018, ^3**^P = 0.0038, ^4**^P = 0.0075 ^5****^P < 0.0001, ^6****^P < 0.0001
Bax	4.05 ± 0.21	2.38 ± 0.1	3.15 ± 0.22	6.00 ± 0.27	^1***^P = 0.0003, ^2*^P = 0.0206 ^3***^P = 0.0001, ^4*^P = 0.0226 ^5****^P < 0.0001, ^6****^P < 0.0001
Bcl-2	1.80 ± 0.1	5.28 ± 0.15	3.90 ± 0.41	1.65 ± 0.1	^1****^P < 0.0001, ^2****^P < 0.0001 ^4**^P = 0.0019, ^5****^P < 0.0001 ^6****^P < 0.0001
Bax/Bcl-2	2.44	0.45	0.81	3.64	--

The validation criteria include glycemic control (HbA1c), hormone profile (FSH, LH and total testosterone), semen profile (count, immotile sperm, motile sperm, rapidly progressive, slowly progressive, motile in place, abnormal form), apoptosis (%), DNA fragmentation (%), gene and protein expressions. All experiments were performed in replicates, and statistical analysis was performed using one-way ANOVA test with post hoc tests.

^*1*^*Normal nondiabetic vs. Abnormal nondiabetic*.

^*2*^*Normal nondiabetic vs. Normal Diabetic*.

^*3*^*Normal nondiabetic vs. Abnormal Diabetic*.

^*4*^*Abnormal nondiabetic vs. Normal Diabetic*.

^*5*^*Abnormal nondiabetic vs. Abnormal Diabetic*.

^*6*^*Normal Diabetic vs. Abnormal Diabetic*.

*P < 0.05, **P < 0.01, ***P < 0.001, ****P < 0.0001.

**Table 3:** Interaction between diabetes and semen status.

Dependent Variable	Factor	F-value	*P*-value	Significant (*P* < 0.05)?
HbA1c	Diabetes	61.552	< 0.0001	Yes
	Semen quality	1.033	0.316	No
	Diabetes × Semen	0.445	0.509	No
Sperm count	Diabetes	0.250	0.620	No
	Semen quality	2.106	0.156	No
	Diabetes × Semen	0.478	0.494	No
Motile sperm	Diabetes	1.682	0.203	No
	Semen quality	20.131	< 0.0001	Yes
	Diabetes × Semen	0.704	0.407	No
FSH	Diabetes	18.42	< 0.0001	Yes
	Semen quality	6.91	0.013	Yes
	Diabetes × Semen	25.87	< 0.0001	Yes
LH	Diabetes	9.84	0.0035	Yes
	Semen quality	1.22	0.278	No
	Diabetes × Semen	9.31	0.0045	Yes
DNA fragmentation	Diabetes	8.332	0.0066	Yes
	Semen quality	3.161	0.084	No
	Diabetes × Semen	3.797	0.059	No
Protein Bax	Diabetes	42.281	< 0.0001	Yes
	Semen quality	7.861	0.0159	Yes
	Diabetes × Semen	116.587	< 0.0001	Yes
Protein Bcl-2	Diabetes	11.544	0.0053	Yes
	Semen quality	7.448	0.0183	Yes
	Diabetes × Semen	162.692	< 0.0001	Yes
Protein Cytochrome-c	Diabetes	25.651	0.0003	Yes
	Semen quality	0.280	0.606	No
	Diabetes × Semen	133.766	< 0.0001	Yes
Protein Caspase-3	Diabetes	28.504	0.0002	Yes
	Semen quality	0.720	0.413	No
	Diabetes × Semen	108.749	< 0.0001	Yes

Two-way ANOVA analysis showing the main effects of diabetes and semen quality on different parameters (semen, diabetes, hormonal, DNA fragmentation, gene and protein expression) at *p* > 0.05.

#### Apoptosis and DNA fragmentation

To explore underlying mechanisms, we assessed sperm apoptosis via flow cytometry and DNA fragmentation (Table 2; Fig. 3C and Fig. 1S). The normal nondiabetic group had a high percentage of live, non-apoptotic sperm (LL parent: 86.4 ± 11.46%). In contrast, all other groups showed a dramatic and significant increase in early apoptotic sperm (UL parent), with the normal diabetic group displaying the highest level (76.4 ± 4.68%, *p < 0.0001 vs.*. control). The abnormal nondiabetic and abnormal diabetic groups also showed significantly elevated early apoptosis (56.2 ± 6.49% and 58.9 ± 8.79%, respectively, *p < 0.0001 vs.*. control). Despite these marked differences in apoptotic markers, no significant differences in DNA fragmentation percentage were observed among the four groups.

#### Gene and protein expression of apoptotic markers

The expression of key apoptotic and inflammatory regulators was then examined (Fig. 3D and E). IL-6 gene expression was the only one significantly higher in the Abnormal diabetic group compared to the Normal diabetic group (*p < 0.05*). Analysis of protein expression via fold-change revealed a distinct pattern of intrinsic pathway activation. The abnormal diabetic group exhibited the highest protein levels of the pro-apoptotic markers Caspase-3 (7.3 ± 0.28, p < 0.0001 vs.. all other groups) and Bax (6.00 ± 0.27, *p* < 0.0001 vs. all other groups), as well as Cyt-C (4.38 ± 0.21, *p < 0.0001 vs.*. all other groups). Conversely, the anti-apoptotic protein Bcl-2 was lowest in the abnormal diabetic group (1.65 ± 0.1) and the normal nondiabetic control (1.80 ± 0.1), while it was significantly elevated in the abnormal nondiabetic (5.28 ± 0.15) and normal diabetic (3.90 ± 0.41) groups. Consequently, the calculated Bax/Bcl-2 ratio, an indicator of apoptotic propensity, was highest in the abnormal diabetic group (3.64), followed by the normal nondiabetic control (2.44), and was lowest in the abnormal nondiabetic (0.45) and normal diabetic (0.81) groups.

### Assessment of the interaction between diabetes and semen quality

To further evaluate whether diabetes status and abnormal semen quality exerted independent or interactive effects on the studied parameters, a two-way ANOVA analysis was performed using diabetes status and semen quality as independent factors, with the diabetes × semen quality interaction term included. The analysis revealed variable effects depending on the measured endpoint. Regarding metabolic and semen parameters, diabetes status showed a significant main effect on HbA1c levels (F = 61.552, *p* < 0.0001), whereas semen quality and the diabetes × semen quality interaction were not significant. No significant effects of diabetes, semen quality, or their interaction were observed for sperm count. In contrast, sperm motility was significantly influenced by semen quality (F = 20.131, *p* < 0.0001), while the interaction between diabetes and semen quality was not significant. Hormonal analysis demonstrated significant effects of both diabetes and semen quality on FSH levels, with a significant diabetes × semen quality interaction (F = 25.87, *p* < 0.0001), indicating that the effect of diabetes on FSH regulation was influenced by semen status. Similarly, LH levels showed significant effects of diabetes (F = 9.84, *p* = 0.0035) and a significant diabetes × semen quality interaction (F = 9.31, *p* = 0.0045). For sperm DNA integrity, diabetes status significantly affected DNA fragmentation (F = 8.332, *p* = 0.0066), while semen quality and the diabetes × semen quality interaction did not reach statistical significance. Importantly, apoptosis-related protein markers showed significant interaction effects between diabetes and semen quality. Bax protein expression demonstrated significant main effects of diabetes (F = 42.281, *p* < 0.0001) and semen quality (F = 7.861, *p* = 0.0159), with a significant diabetes × semen quality interaction (F = 116.587, *p* < 0.0001). Similar interaction patterns were observed for Bcl-2 protein (F = 162.692, *p* < 0.0001), cytochrome-c protein (F = 133.766, *p* < 0.0001), and caspase-3 protein (F = 108.749, *p* < 0.0001). Overall, these findings indicate that the interaction between diabetes and abnormal semen status was particularly evident in hormonal regulation and mitochondrial apoptosis-related pathways, whereas conventional semen parameters showed mainly independent effects of semen status without significant statistical interaction.

### The correlation between semen quality, hormonal levels, and apoptotic markers in all patient groups

**Fig. 4:**
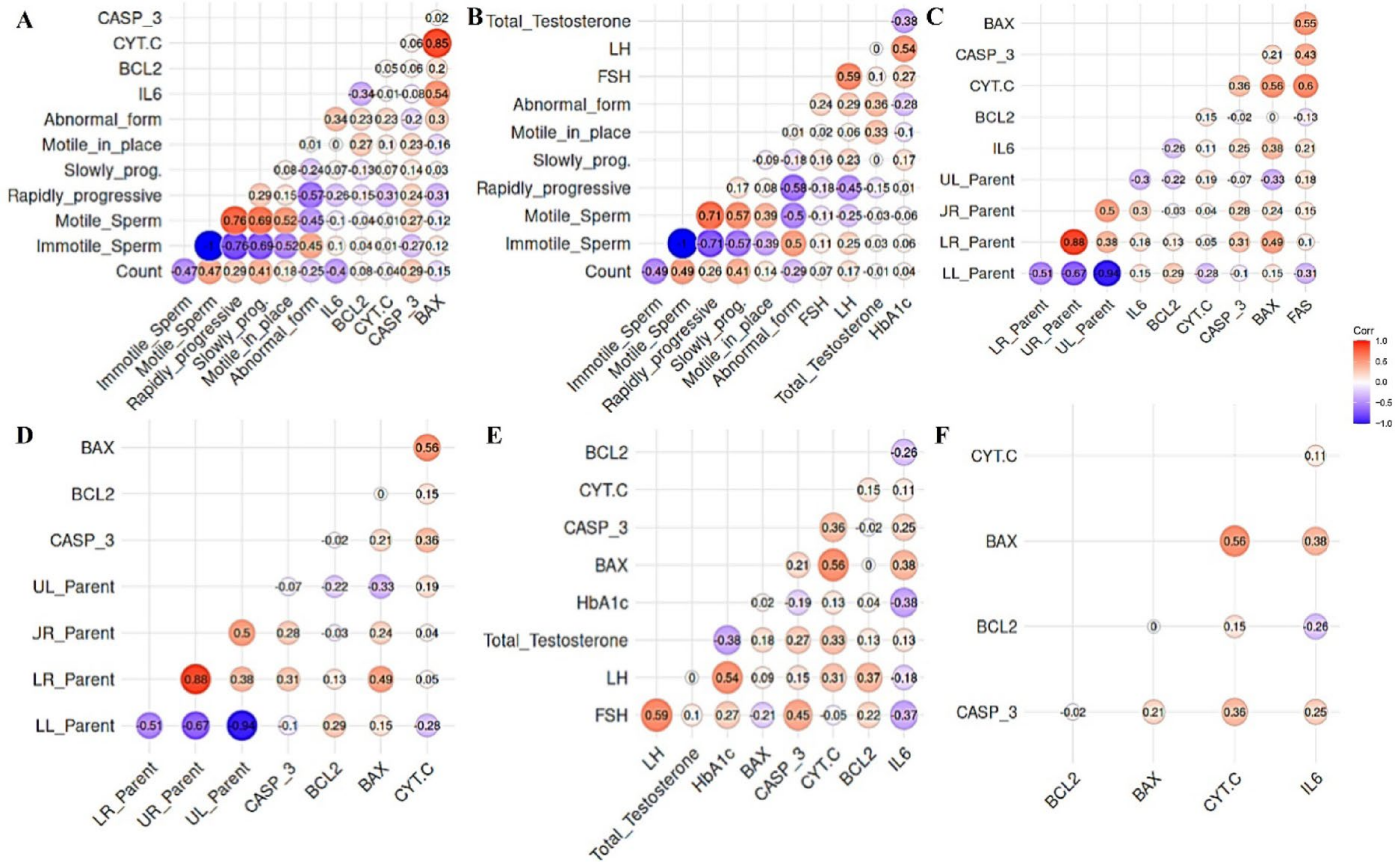
Correlation heatmaps of semen quality, hormonal levels, apoptotic markers, gene expression, and protein concentration. Red indicates positive correlation, blue indicates negative correlation, and circle size represents correlation strength (larger circle = stronger correlation). (**A**) Sperm quality vs. apoptotic biomarkers. (**B**) Semen profile vs. hormones. (**C**) Apoptosis vs. gene expression. (**D**) Apoptosis vs. protein concentration. (**E**) Apoptosis vs. hormone profile, and (**F**) Gene expression vs. protein content. Abbreviations: Bax, Bcl-2, Casp-3, Cyt-C, FAS, IL6, LH, FSH, HbA1c, LL/ LR/ UR/ UL Parent (apoptotic stages).

Detailed analysis of the correlations between semen quality, hormonal levels, and apoptotic markers has been performed using heatmap analysis (Fig. 4). The figure utilizes a color scale where red indicates a positive correlation, blue indicates a negative (inverse) correlation, and the size of the circle represents the strength of that relationship. A series of six heatmaps was generated to examine pairwise correlations among physical sperm parameters, endocrine markers (LH, FSH, total testosterone, HbA1c), apoptotic biomarkers (Bax, Bcl-2, Casp-3, Cyt-C, IL-6), and gene/protein expression levels. Figure 4A illustrates the relationships between physical sperm characteristics and markers of programmed cell death. As expected, immotile and motile sperm showed a perfect inverse relationship, confirming internal consistency. Rapid progressive sperm were strongly negatively correlated with immotile sperm (-0.76) and with slow progressive sperm (-0.69). The pro-apoptotic marker Bax demonstrated moderate positive correlations with abnormal sperm morphology (0.32 and 0.19, respectively). Sperm count was negatively correlated with almost all apoptotic markers, most notably with Bax (-0.29) and Casp-3 (-0.29). Figure 4B presents the endocrine relationships with sperm health and the metabolic marker HbA1c. Luteinizing hormone (LH) showed a strong positive correlation with follicle-stimulating hormone (FSH) (0.59) and with total testosterone (0.54). Total testosterone was negatively correlated with abnormal sperm forms (-0.38) but showed a negligible correlation with motility (*r* = 0.06). HbA1c was negatively correlated with total testosterone (-0.28) and with sperm count (-0.04). As shown in Fig. 4C, a very strong negative correlation (-0.94) was observed between LL-Parent (viable cells) and UL-Parent (necrotic cells). Cyt-C and Bax showed strong positive correlations with each other (0.56) and with Casp-3 (0.36 and 0.21, respectively). Figure 4D correlates apoptotic stages with protein concentrations involved in cell death. A strong positive correlation was observed between LR-Parent and UR-Parent (0.88). Bax protein levels showed a moderate positive correlation with Cyt-C (0.56) and with Casp-3 (0.21). The LL-Parent group demonstrated negative correlations with all major apoptotic proteins, including Bax (-0.15) and Cyt-C (-0.28). Figure 4E examines relationships between hormonal levels, metabolic markers, and apoptotic proteins. A strong positive correlation was found between LH and FSH (0.59). Total testosterone showed a strong positive correlation with LH (0.54) and a notable negative correlation with HbA1c (-0.38). HbA1c was negatively correlated with Casp-3 (-0.19) and IL6 (-0.38) but showed a very weak positive correlation with Bax (0.02). Bax was strongly positively correlated with Cyt-C (0.56). Casp-3 showed its strongest positive correlations with Cyt-C (0.43) and IL6 (0.25). Bcl-2 was negatively correlated with IL6 (-0.26) while maintaining a moderate positive correlation with LH (0.37). FSH demonstrated a moderate positive correlation with Casp-3 (0.45) and a negative correlation with IL-6 (-0.37). Total testosterone displayed positive correlations with Cyt-C (0.33), Casp-3 (0.27). Figure 4F illustrates the relationships between apoptotic markers at the gene and protein levels. Bax showed strong positive correlations with Cyt-C (0.56). Casp-3 was positively correlated with Cyt-C (0.36). Bcl-2 showed negative correlations with IL-6 (-0.26). IL6 had a moderate positive correlation with Bax (0.38) and Casp-3 (0.25). Across all figures, consistent patterns emerged. Total testosterone was negatively correlated with abnormal sperm morphology (Fig. 2) and positively correlated with Casp-3 (Fig. 4E). FSH and LH were strongly correlated (0.59, Fig. 4E), and FSH showed a moderate positive correlation with Casp-3 (0.45, Fig. 4E). Referring to Fig. 4A, Casp-3 was negatively correlated with sperm count (-0.29) and with rapidly progressive sperm (-0.15). HbA1c was strongly negatively correlated with total testosterone (-0.38, Fig. 4E), and total testosterone was linked to lower abnormal forms (-0.38, Fig. 4B). Bax and Cyt-C consistently showed a strong positive correlation (0.56) across gene, protein, and hormonal profiles (Fig. 4C-E). In Fig. 4A, these markers were correlated with increased abnormal forms (BAX: 0.32) and decreased sperm count (Bax: -0.29). In Fig. 4E, LH and FSH were positively correlated with BAX and Cyt-C.

## Discussion

The present study provides a comprehensive investigation into the complex interplay between diabetes mellitus and male infertility, extending beyond simple validation of patient groups to elucidate potential molecular mechanisms. By classifying patients into four distinct groups, normal nondiabetic, abnormal nondiabetic, normal diabetic, and abnormal diabetic, the study was able to dissect the individual and enhanced effects of hyperglycemia and abnormal semen parameters on reproductive function, apoptosis, and associated molecular pathways. The initial validation phase confirmed the appropriate classification of patients into diabetic and nondiabetic groups, as well as fertile and abnormal infertile groups. The significantly elevated HbA1c levels in diabetic patients showed poor glycemic control, consistent with the diagnostic criteria for diabetes mellitus^21^. The observed hormonal alterations in diabetic patients, including elevated FSH and LH coupled with reduced testosterone, align with the well-documented phenomenon of “diabetic hypogonadism^22^. Several studies have demonstrated that diabetes can disrupt the hypothalamic-pituitary-gonadal (HPG) axis, leading to secondary hypogonadism^22,23^. The mechanisms proposed include oxidative stress-induced Leydig cell dysfunction^5,14,24,25^, insulin resistance at the pituitary level^25,26^, and advanced glycation end-product (AGE) accumulation in testicular tissue^5^,^27^.

In our cohort, the diabetic groups showed alterations in gonadotropin profiles, particularly FSH and LH changes, supporting the presence of endocrine dysregulation despite the absence of a statistically significant testosterone difference among the four subgroups^28,29^. Furthermore, testosterone regulation is influenced by compensatory feedback mechanisms. In some diabetic individuals, increased gonadotropin stimulation may partially preserve testosterone production despite underlying Leydig cell dysfunction.

The diabetic group in the validation study exhibits a higher BMI than the non-diabetic group, but the difference was not statistically significant. An interesting study revealed that a high BMI is considered a predisposing factor for diabetes, especially for those with a BMI over 35 kg/m^2^, who had an increased risk of developing type II diabetes^30^. Although BMI is a good indicator of type II diabetes, some researchers suggest that other measurement tools are more useful for this prediction^31^. While this finding may initially seem counterintuitive given the association between obesity and type 2 diabetes, it is important to note that our diabetes may include those patients with well-managed diabetes. Alternatively, this could also reflect the phenomenon of “diabetic cachexia”^32–34^ or unintentional weight loss associated with poor glycemic control^35^.

The validation of abnormal infertile patients revealed the expected impairments in semen parameters, particularly the dramatic reduction in rapidly progressive sperm and increased abnormal morphology. These parameters are critical determinants of male fertility potential, as rapid progressive motility is essential for sperm transport through the female reproductive tract and oocyte penetration^36^. Notably, the hormonal profile in abnormal infertile patients did not differ significantly from fertile controls, suggesting that the observed semen abnormalities in this group are likely due to primary testicular factors rather than endocrine dysfunction^37,38^.

The four-group classification revealed that the combination of diabetes and abnormal semen parameters (abnormal diabetic group) generally resulted in the most severe reproductive phenotype, suggesting an enhanced combined effect. The hormonal analysis revealed distinct patterns across groups. The Abnormal nondiabetic group showed elevated FSH and LH with preserved testosterone, a pattern characteristic of compensated Sertoli and Leydig cell dysfunction or primary testicular failure^39,41^. Elevated FSH is particularly indicative of Sertoli cell dysfunction and is commonly observed in patients with impaired spermatogenesis^42^. The normal diabetic group exhibited even higher FSH and LH levels, supporting the concept that diabetes independently disrupts the HPG axis at multiple levels. Hyperglycemia-induced oxidative stress has been shown to directly damage Leydig cells, reducing testosterone production and consequently removing negative feedback on gonadotropin secretion^43,44^. Additionally, insulin signaling plays a direct role in hypothalamic GnRH secretion^45^, and insulin resistance^46^ or deficiency can impair this process^47,48^. The abnormal diabetic group showed an intermediate FSH level that was significantly lower than the Normal diabetic group but higher than controls. This intriguing finding may reflect a complex interplay between metabolic and testicular factors, potentially involving differential regulation of inhibin B production^42^ or altered pituitary sensitivity to GnRH^49^.

The semen analysis provided compelling evidence for enhanced combined effect damage in the abnormal diabetic group. While both abnormal nondiabetic and abnormal diabetic groups showed impaired motility parameters, the abnormal diabetic group demonstrated the lowest rapid progressive motility, significantly worse than even the abnormal nondiabetic group. This finding aligns with previous reports that diabetes exacerbates existing fertility problems^50,51^. Diabetes affects sperm motility through multiple mechanisms. Hyperglycemia leads to increased oxidative stress in seminal plasma, with elevated reactive oxygen species (ROS) directly damaging sperm membranes and mitochondrial DNA^52,53^. Spermatozoa are particularly vulnerable to oxidative damage due to their limited antioxidant defenses and high content of polyunsaturated fatty acids in their membranes^54,56^. Furthermore, diabetes-induced impairment of mitochondrial function directly affects the energy production required for flagellar movement^57^. The high percentage of abnormal sperm morphology across all groups compared to controls indicates that both diabetes and primary testicular factors independently affect spermatogenesis^57^. The abnormal nondiabetic group showed the highest morphological abnormalities, suggesting that in our group, primary testicular factors may have a stronger impact on sperm structure than diabetes alone^58^.

The apoptosis analysis provided critical insights into the cellular mechanisms underlying the observed fertility impairments. The dramatic reduction in live, non-apoptotic sperm (LL parent) in all groups compared to controls indicates that both diabetes and primary infertility are associated with increased germ cell apoptosis^59^. The normal diabetic group exhibited the highest level of early apoptotic sperm (UL parent), even higher than the abnormal nondiabetic group. Annexin V positivity primarily indicates early externalization of phosphatidylserine, which is considered an early marker of apoptosis-like membrane alteration, while PI exclusion indicates preservation of membrane integrity. Therefore, Annexin V/PI analysis may detect early cellular stress or mitochondrial-related apoptotic signaling before the development of functional impairment manifested as reduced mobility or abnormal morphology. In addition, apoptosis-related changes detected by flow cytometry may represent partial or abortive apoptotic processes, residual molecular alterations originating during spermatogenesis, or mitochondrial stress responses rather than complete sperm elimination. This interpretation is consistent with our findings that diabetes induced apoptotic changes even in the normal diabetic group, while more extensive molecular alterations were observed when diabetes coexisted with abnormal semen status. The preservation of motility and morphology in the normal diabetic group does not exclude the presence of early cellular damage. Sperm functional parameters represent the outcome of multiple cellular processes, whereas apoptosis-related markers may identify earlier molecular disturbances that precede measurable impairment. Similar dissociations have been reported in which molecular indicators of cellular stress are altered before conventional semen parameters become abnormal. Therefore, we interpret the elevated Annexin V/PI-positive population in the Normal diabetic group as evidence of early diabetes-associated sperm cellular stress rather than direct evidence of impaired fertility potential. In contrast, the Abnormal diabetic group showed a broader phenotype characterized by impaired semen parameters together with increased apoptotic pathway activation, suggesting progression from early molecular alterations to functional sperm impairment. This finding suggests that diabetes is a potent inducer of apoptosis in the testis, independent of baseline semen quality^9^. Experimental models have consistently shown that diabetes increases germ cell apoptosis through multiple pathways, including hyperglycemia-induced oxidative stress, activation of the intrinsic mitochondrial pathway, and disruption of Sertoli cell-germ cell interactions^19,60,61^. Despite the marked differences in apoptotic markers, DNA fragmentation did not differ significantly across groups. This apparent discrepancy may be explained by the fact that DNA fragmentation represents a later stage of cell damage, and the apoptotic cells detected by flow cytometry may be cleared before significant DNA fragmentation occurs^62^. Alternatively, different apoptotic pathways may be activated, some of which do not necessarily result in immediate DNA strand breaks^63^. Activation of the intrinsic mitochondrial apoptotic pathway was based on the coordinated increase in several mitochondrial-associated apoptotic markers, including Bax, cytochrome c, caspase-3, and the Bax/Bcl-2 ratio, together with the increased proportion of Annexin V-positive spermatozoa. The absence of significant differences in DNA fragmentation among the study groups does not necessarily contradict activation of apoptosis-related signaling. First, phosphatidylserine externalization (Annexin V positivity) and mitochondrial dysfunction are considered early apoptotic events, whereas detectable DNA fragmentation represents the necrotic stage of cellular damage^64^. Thus, spermatozoa may exhibit activation of apoptosis-associated pathways without developing measurable DNA strand breaks. Second, mature sperm chromatin is highly compacted by protamines, making it relatively resistant to DNA fragmentation compared with somatic cells. Consequently, mitochondrial apoptotic signaling and membrane alterations may occur independently of, or precede, detectable DNA fragmentation^65,66^. Importantly, we do not claim that mature spermatozoa undergo complete classical apoptosis. Rather, our findings support the presence of apoptosis-like, or abortive apoptotic processes characterized by activation of mitochondrial stress signaling pathways^67,70^.

The gene and protein expression analyses provided mechanistic depth to the apoptotic findings. The significantly elevated IL-6 gene expression examined in the purified motile spermatozoa isolated from the abnormal diabetic group compared to normal diabetic group suggests an enhanced inflammatory state when both conditions coexist. IL-6 is a pro-inflammatory cytokine that has been implicated in diabetes complications and male infertility^71,73^. Elevated IL-6 in seminal plasma has been associated with reduced sperm motility and increased leukocyte infiltration in the reproductive tract^74,75^. Thus, this study suggested the necessity of employing highly purified sperm populations and cell-specific validation approaches to confirm the cellular origin of these signals for all future studies.

Moreover, the protein expression data revealed a clear pattern of intrinsic (mitochondrial) pathway activation, particularly in the abnormal diabetic group. The significantly elevated levels of Bax, cytochrome c, and caspase-3 in this group, coupled with the lowest Bcl-2 expression, resulted in the highest Bax/Bcl-2 ratio, indicating a strong pro-apoptotic shift. These findings are consistent with activation of mitochondrial stress and apoptosis-associated signaling. Based on our experimental design, these markers reflect residual molecular footprints of apoptotic or stress-related events that occurred during germ-cell development in the testis or during epididymal transit. Such alterations may subsequently be retained in mature sperm and serve as indicators of impaired spermatogenesis and sperm quality.

Regarding the observation that some mRNA gene levels did not differ significantly despite marked alterations at the protein level. Several biological and technical factors may account for these apparent observations. First, post-transcriptional regulatory mechanisms may contribute substantially to the observed findings. Selective mRNA degradation altered translational efficiency, RNA-binding proteins, microRNA-mediated regulation, and differential protein stability can all uncouple transcript and protein levels. Apoptosis-related proteins such as Bax, Bcl-2, cytochrome-c, and caspase-3 are also subject to extensive post-translational regulation, including protein activation, mitochondrial release, proteolytic processing, and altered degradation rates. Therefore, substantial changes in protein abundance and activity can occur without parallel changes in mRNA expression^76,77^. Second, the apoptotic response observed in diabetic and infertile patients may primarily reflect activation of pre-existing protein pools rather than transcriptional induction. For example, mitochondrial stress can trigger cytochrome-c release and caspase activation through post-translational mechanisms that do not require increased gene transcription^78,80^. This interpretation is supported by the significant increases in apoptotic protein markers, elevated Bax/Bcl-2 protein ratio, and flow-cytometric evidence of increased apoptosis observed in our study. Therefore, our conclusions regarding apoptotic pathway activation are based primarily on the concordant protein-expression and functional apoptosis findings rather than on gene-expression changes alone.

The Bax/Bcl-2 ratio is considered a critical determinant of cell fate, with higher ratios promoting mitochondrial outer membrane permeabilization and subsequent activation of the caspase cascade^81^. This pattern suggests that the combination of diabetes and abnormal semen parameters synergistically activates the mitochondrial apoptotic pathway.

Regarding the Bax/Bcl-2 gene expression, it did not differ significantly among the study groups; it was intended to provide a complementary assessment of transcriptional regulation of apoptosis-related markers rather than to serve as the primary evidence for activation of the apoptotic pathway. Whereas alterations were observed at the protein level due to Bax protein expression increased, Bcl-2 protein expression decreased, and consequently the resulting Bax/Bcl-2 protein ratio was markedly elevated in the abnormal diabetic group. Therefore, the conclusions regarding activation of apoptotic signaling are based principally on the protein expression data and the associated Bax/Bcl-2 protein ratio, together with the increased cytochrome c and caspase-3 protein levels.

Moreover, the observed higher Bax/Bcl-2 ratio in the normal group may reflect biological variability among human sperm samples, differences in basal apoptotic regulation, and variation in individual sperm quality parameters. It is also important to note that mature spermatozoa naturally contain apoptosis-related proteins as part of normal cellular regulation, and the presence of these proteins does not necessarily indicate pathological apoptosis. The statistical analysis was performed using biological replicates, and the interpretation was based on the integrated pattern of apoptosis-related markers rather than the Bax/Bcl-2 ratio alone.

Diabetes-induced oxidative stress can directly activate Bax and inhibit Bcl-2 through multiple mechanisms, including JNK signaling^82^ and p53 activation^83,84^. Concurrently, pre-existing testicular pathology may sensitize germ cells to these pro-apoptotic signals, lowering the threshold for apoptosis induction^85–88^. The unexpected finding of elevated Bcl-2 protein in the abnormal nondiabetic and normal diabetic groups, despite increased apoptosis, may represent a compensatory anti-apoptotic response. Chronic stress conditions can initially upregulate protective mechanisms before they are overwhelmed^89–91^. The fact that this compensation fails in the abnormal diabetic group, where Bcl-2 is lowest despite the highest apoptotic load, underscores the severity of the combined insult.

Interestingly, the absence of significant differences in DNA fragmentation among the study groups does not necessarily contradict activation of apoptosis-related signaling. First, phosphatidylserine externalization (Annexin V positivity) and mitochondrial dysfunction are considered early apoptotic events, whereas detectable DNA fragmentation represents the necrotic stage of cellular damage^64^. Thus, spermatozoa may exhibit activation of apoptosis-associated pathways without developing measurable DNA strand breaks. Second, mature sperm chromatin is highly compacted by protamines, making it relatively resistant to DNA fragmentation compared with somatic cells. Consequently, mitochondrial apoptotic signaling and membrane alterations may occur independently of, or precede, detectable DNA fragmentation^65,66^. Importantly, we do not claim that mature spermatozoa undergo complete classical apoptosis. Rather, our findings support the presence of apoptosis-like or abortive apoptotic processes characterized by activation of mitochondrial stress signaling pathways^67–70^.

The present study demonstrates significant correlations between semen quality parameters, hormonal levels, and apoptotic markers, providing important insights into the molecular mechanisms underlying male infertility. The strong positive correlations observed between Bax, Cyt-C, and Casp-3 across gene, protein, and hormonal profiles align with the well-established intrinsic mitochondrial apoptotic pathway^92^. In this pathway, under stress conditions, Bax undergoes a conformational change and translocates to the mitochondrial outer membrane, leading to cytochrome c release and subsequent Casp-3 activation, ultimately resulting in programmed cell death. The finding that these markers are positively correlated with abnormal sperm morphology and negatively correlated with sperm count supports the critical role of apoptosis in regulating spermatogenic output. Indeed, it has been established that up to 75% of germ cells undergo apoptosis during normal spermatogenesis to eliminate defective cells, and dysregulation of this process is implicated in male infertility^92^.

Activation of mitochondrial stress and apoptosis-associated signaling reflect residual molecular footprints of apoptotic or stress-related events that occurred during germ-cell development in the testis or during epididymal transit. Such alterations may subsequently be retained in mature sperm and serve as indicators of impaired spermatogenesis and sperm quality. The observed increase in Annexin V-positive spermatozoa suggests that at least some apoptosis-like membrane and mitochondrial changes remain detectable at the ejaculated sperm stage.

The endocrine relationships observed in this study reveal a complex interplay between the hypothalamic-pituitary-gonadal (HPG) axis and apoptotic signaling. The strong positive correlation between LH and FSH and their associations with both testosterone and apoptotic markers suggest that gonadotropins may influence spermatogenesis through multiple pathways. Notably, the positive correlation between FSH and Casp-3, combined with the negative correlation between Casp-3 and sperm count, indicates that elevated gonadotropin levels might be associated with increased apoptotic signaling. This finding is consistent with recent research demonstrating that diabetes-induced disruption of the HPG axis impairs testicular function and spermatogenesis^57^. Furthermore, the observation that total testosterone shows a negative correlation with abnormal sperm morphology while maintaining positive correlations with apoptotic markers suggests that testosterone plays a dual role in spermatogenesis supporting proper morphological development while also participating in the regulation of germ cell elimination.

The two-way ANOVA results demonstrated that the interaction between diabetes and semen status was outcome-dependent. For conventional semen parameters, significant effects were mainly observed for semen status, particularly for sperm motility parameters, whereas the diabetes × semen Status interaction was not statistically significant for most semen characteristics. This indicates that the effect of diabetes on some basic semen parameters may not exceed an additive pattern. However, significant Diabetes × Semen Status interactions were observed for several mechanistic endpoints. Specifically, significant interaction effects were detected for hormonal parameters including FSH and LH, as well as apoptosis-related molecular markers including Bax, Bcl-2, cytochrome-c, and caspase-3 protein expression. These findings indicate that the impact of diabetes on endocrine regulation and mitochondrial apoptotic signaling is modified by semen status, supporting an enhanced combined effect at the molecular level. The metabolic findings, particularly the negative correlations between HbA1c and both total testosterone and Casp-3, highlight the potential impact of glycemic control on male reproductive health. These results align with growing evidence that diabetes mellitus adversely affects male reproductive function through multiple mechanisms, including oxidative stress, endocrine disruption, and increased apoptosis^57,93^. The negative correlation between HbA1c and total testosterone suggests that poor glycemic control may suppress testosterone production, which could indirectly contribute to poorer sperm morphology and reduced fertility potential. This finding is particularly relevant given that approximately 90% of diabetic men experience varying degrees of testicular dysfunction^57^, and hyperglycemia has been shown to disrupt the balance of oxidants and antioxidants, leading to activation of the intrinsic apoptotic pathway in germ cells^94,95^. The consistency of correlations across gene expression, protein concentration, and hormonal profiles provides strong biological validity to these findings. The synchronization between Bax and Cyt-C and their consistent association with poor semen quality parameters supports the central role of mitochondrial dysfunction in male infertility. Moreover, the negative correlations observed between Bcl-2 (an anti-apoptotic marker) and inflammatory markers such as IL6 suggest that inflammation may promote cell death by suppressing protective pathways. Though, the correlation analysis and heatmap clustering demonstrated coordinated relationships between impaired semen quality, hormonal alterations, inflammatory/apoptotic markers, and mitochondrial pathway activation. The abnormal diabetic group consistently showed the most pronounced molecular alterations compared with the other groups, supporting the biological relevance of the observed interaction pattern. These findings collectively suggest that targeting oxidative stress and apoptotic pathways may represent a potential therapeutic strategy for managing male infertility, particularly in the context of metabolic disorders such as diabetes^57,93^. Future studies should focus on evaluating the efficacy of antioxidant therapies and glycemic control interventions in improving semen quality by modulating these apoptotic pathways.

These findings have several important clinical implications. First, they highlight the need for comprehensive fertility evaluation in diabetic men, even in the absence of overt semen abnormalities, as diabetes alone can induce significant testicular apoptosis and hormonal dysfunction. Second, the enhanced combined damage observed in the abnormal diabetic group suggests that diabetic men with pre-existing fertility factors may require more aggressive management, including optimization of glycemic control and consideration of early fertility preservation^96,97^. The demonstration that diabetes activates the intrinsic apoptotic pathway in germ cells opens potential therapeutic avenues. Antioxidant therapies, such as vitamin E, coenzyme Q10, and N-acetylcysteine, have shown promise in reducing oxidative stress and improving sperm parameters in diabetic men^98,99^. More targeted approaches, such as inhibitors of the mitochondrial permeability transition pore or Bax inhibitors, may represent future therapeutic strategies, though clinical data are currently lacking^100^.

This study has suggested limitations that should be acknowledged. The relatively small sample size in each subgroup due to the complex social issue deeply embedded in cultural norms limits the generalization of our findings. The cross-sectional design precludes determination of causality, and longitudinal studies would be valuable to assess the temporal relationship between diabetes progression and fertility decline. Additionally, we did not assess seminal oxidative stress markers or AGE levels, which would provide direct evidence for the proposed mechanisms. Therefore, some future perspectives are recommended. First, larger, multicenter cohorts’ studies are recommended to include detailed characterization of diabetes type, duration, and treatment. Second, mechanistic studies using in-vitro models could elucidate the specific molecular pathways by which hyperglycemia induces germ cell apoptosis. Also, studies employing highly purified sperm populations and cell-specific validation approaches will be necessary to confirm the cellular origin of these signals. Studies can be performed using testicular tissue, epididymal sperm, mitochondrial membrane potential assays, and additional functional analyses will be required to determine the precise temporal origin and biological significance of these molecular alterations. Finally, interventional trials are needed to determine whether optimizing glycemic control or administering antioxidant therapy can reverse or prevent the reproductive damage associated with diabetes.

## Conclusion

In conclusion, this study demonstrates that diabetes mellitus is associated with significant reproductive dysfunction, characterized by hormonal imbalances, impaired semen parameters, and increased germ cell apoptosis. When diabetes coexists with pre-existing semen abnormalities, enhanced combined damage occurs, particularly affecting sperm motility and activating the intrinsic mitochondrial apoptotic pathway. The elevated Bax/Bcl-2 ratio, cytochrome c release, and caspase-3 activation in the abnormal diabetic group provide a molecular explanation for the enhanced germ cell loss observed in these patients. These findings underscore the importance of reproductive health monitoring in diabetic men and suggest that targeting the mitochondrial apoptotic pathway may offer therapeutic potential for preserving fertility in this population.

## Materials and methods

### Study design and patient enrollment

This study was designed to investigate the impact of diabetes mellitus on male reproductive function and to elucidate the molecular mechanisms underlying diabetes-associated infertility. The study was conducted at the Benha Fertility Center, Benha University, and the Centre of Excellence Helwan Structural Biology Research (HSBR), Faculty of Pharmacy, Capital University (formerly Helwan University) over a period of 2024–2025. All experimental protocols were approved by the Faculty of Pharmacy, Capital University (formerly Helwan University) Ethics Committee (Approval No:02H2022), and written informed consent was obtained from all participants prior to enrollment, in accordance with the Declaration of Helsinki.

### Patient stratification and group allocation

Sample size and power analysis were calculated using G*Power version 3.1.9.4^101^. The study evaluated multiple outcomes, including hormonal parameters, semen characteristics, apoptosis markers, gene expression levels, and protein expression levels. The calculation was based primarily on the main clinical outcomes (semen and hormonal parameters) and assumed a large effect size (Cohen’s d = 1.1), α = 0.05, power = 0.80, and an allocation ratio of 1.16, resulting in a required total sample size of approximately 39 participants. To minimize errors, the statistical analyses were guided by predefined biological hypotheses regarding the relationship between obesity, male infertility, and the investigated molecular pathways. Accordingly, the selected molecular markers (IL-6, Caspase-3, Cytochrome c, Bax, and Bcl-2) were not independent exploration variables but mechanistically related components of the same biological pathway. Furthermore, the interpretation of the results was therefore based on the overall consistency of findings across multiple levels of evidence, including hormonal alterations, semen characteristics, flow-cytometric apoptosis measurements, and corresponding gene and protein expression profiles, rather than on isolated statistically significant observations. Where appropriate, correction for multiple comparisons was considered/applied. The sample size was determined primarily to detect differences in the main clinical outcomes (semen parameters and hormonal measurements). For the gene-expression and protein-expression analyses, a separate exploratory subgroup analysis was planned. Based on published evidence demonstrating larger biological differences for molecular biomarkers, a target effect size of Cohen’s d = 1.5 was assumed. The unequal subgroup sizes reflect the natural distribution of eligible participants during recruitment rather than a priori stratified allocation. All the molecular analyses (gene and protein expression of apoptotic and inflammatory markers). Samples were analyzed in technical triplicates to ensure analytical reliability and reproducibility. The mean value of the triplicate measurements for each sample was used for statistical comparison among groups. The number of biological samples included in each group was sufficient to allow statistical evaluation of gene and protein expression data using one-way ANOVA followed by appropriate post hoc analysis. The triplicate measurements were considered technical replicates and were not treated as independent biological observations; therefore, statistical inference was based on the biological replicates (individual samples).The sample size per group was derived using the formula for independent samples t-tests^102^:

$$\:n=\frac{2\left(\frac{Z\alpha\:}{2}+Z\beta\:\right)^2}{d^2}$$

Data were assessed for normality using the Shapiro-Wilk test. In instances where non-normal distribution was confirmed, the Mann-Whitney U test was employed. By utilizing a total sample of $*N* = 39$, the study accounts for the slightly lower asymptotic relative efficiency of non-parametric testing, ensuring sufficient power to detect large effect sizes. The significant differences observed in molecular outcomes indicate that the experimental design had adequate sensitivity to detect group-related changes in gene and protein expression.

#### Initial validation cohort

For the initial validation of patient selection criteria, participants were stratified into two comparative analyses:

### Diabetes validation

Participants were divided into nondiabetic patients (*n* = 21; healthy volunteers with no history of diabetes and normal fasting blood glucose < 100 mg/dL and HbA1c < 5.7%) and diabetic patients (*n* = 18; patients with an established diagnosis of diabetes mellitus for at least 5 years, with HbA1c ≥ 6.5% at enrollment according to American Diabetes Association (ADA) 2021^103^. The abnormal diabetic patients were based on persistent abnormalities rather than a single blood examination.

### Infertility validation

Participants were divided into normal fertile patients (*n* = 20; healthy volunteers with normal semen parameters according to WHO criteria and confirmed fertility^104^ and abnormal infertile patients (*n* = 19; patients presenting with infertility for at least 12 months of regular unprotected intercourse, with at least two abnormal semen parameters according to WHO criteria 6th edition. The WHO 2021 reference limits used for classification includes, Sperm concentration/ml: ≥16 million/ejaculate, total motility: ≥42%, progressive motility: ≥30%, normal morphology: ≥4%. Participants were classified as abnormal only when two or more semen variables were below the WHO reference thresholds^104^. Men were considered “abnormal infertile” if they had a clinical history of infertility (failure to achieve conception after at least 12 months of regular unprotected intercourse) and exhibited at least two abnormal semen parameters based on WHO reference limits. To improve classification accuracy, semen analysis was performed in triplicate at intervals of at least two weeks.

#### Four-group mechanistic study

Following validation, participants were further stratified into four distinct groups for mechanistic analysis: Group 1: Normal nondiabetic group (*n* = 10): Healthy volunteers with normal semen parameters, normal HbA1c (< 5.7%), and no history of diabetes or fertility problems. Group 2: Abnormal nondiabetic group (*n* = 11): Patients with abnormal semen parameters but normal HbA1c (< 5.7%) and no history of diabetes. Group 3: Normal diabetic group (*n* = 10): Diabetic patients (HbA1c ≥ 6.5%) with normal semen parameters. Group 4: Abnormal diabetic group (*n* = 8): Diabetic patients (HbA1c ≥ 6.5%) with abnormal semen parameters.

### Inclusion and exclusion criteria

#### Inclusion criteria

All participants were required to meet the following criteria: male gender; age between 20 and 50 years; body mass index (BMI) between 18.5 and 35 kg/m²; for diabetic groups: documented diagnosis of diabetes mellitus for at least 5 years; for nondiabetic groups: fasting blood glucose < 100 mg/dL and HbA1c < 5.7%; for fertile groups: confirmed paternity within the past 2 years or normal semen parameters with no history of infertility; for infertile groups: at least 12 months of unprotected intercourse without conception and abnormal semen parameters.

#### Exclusion criteria

Participants were excluded if they met any of the following criteria: history of genitourinary tract infection within the preceding 3 months; varicocele (grade II or higher); cryptorchidism or testicular torsion; genetic causes of infertility; obstructive azoospermia; history of chemotherapy or radiotherapy; use of medications affecting spermatogenesis; chronic systemic diseases other than diabetes; history of alcohol abuse or illicit drug use; occupational exposure to reproductive toxins.

### Clinical and anthropometric assessment

All participants underwent comprehensive clinical evaluation, including detailed medical history with emphasis on diabetes duration; fertility history, including duration of infertility; physical examination, including assessment of secondary sexual characteristics; anthropometric measurements, including height (cm), weight (kg), and BMI calculated as weight/height² (kg/m²); and blood pressure measurement.

### Sample collection

#### Blood samples

Venous blood samples (10 mL) were collected from each participant after an overnight fast (8–12 h) between 8:00 and 10:00 AM to minimize circadian variation. Blood was divided into 2 mL in EDTA tubes for HbA1c analysis and 8 mL in plain tubes for serum separation. Blood was allowed to clot at room temperature for 30 min, then centrifuged at 3000 rpm for 15 min at 4 °C. Serum was aliquoted into sterile Eppendorf tubes and stored at -80 °C until analysis.

#### Semen samples

Semen samples were collected by masturbation into sterile, wide-mouth containers after a recommended abstinence period of 3–5 days. Participants were instructed to avoid alcohol, caffeine, and any medications for 72 h prior to collection. Samples were transported to the laboratory within 30 min of collection and maintained at 37 °C during transport and processing. Each sample was divided into routine semen analysis (performed immediately), sperm apoptosis assessment by flow cytometry (processed within 1 h), DNA fragmentation analysis (processed within 2 h), and molecular analyses (sperm pellets stored at -80 °C for gene and protein expressions). All molecular assays were not performed on unprocessed liquefied semen. Semen samples were first subjected to density-gradient centrifugation (45%/100%), followed by washing and swim-up preparation. This procedure was specifically employed to enrich for motile spermatozoa and to remove the majority of leukocytes, round cells, somatic cells, cellular debris, and other contaminants before downstream molecular analyses.

### Laboratory analyses

#### Glycemic assessment

Glycated hemoglobin (HbA1c) was measured in whole blood samples using high-performance liquid chromatography (HPLC) on a Bio-Rad D-10™ Hemoglobin Testing System (Bio-Rad Laboratories, Hercules, CA, USA) according to the manufacturer’s instructions^105^. The separated hemoglobin fractions passed through a flow cell and were measured at a wavelength of 415 nm. The percentage of HbA1c was calculated by dividing the AUC of the HbA1c peak by the AUC of all hemoglobin peaks.

#### Hormonal assays

Serum hormone levels were measured using commercially available enzyme-linked immunosorbent assay (ELISA) kits according to manufacturers’ protocols.

##### Follicle-stimulating hormone (FSH)

Serum FSH was measured using Human FSH ELISA kit (Catalog No. BC1035, BioCheck, Inc., Foster City, California, USA)^106^. The absorbance (OD) of all samples was determined at 450 nm using (BioTek ELx800, BioTek Instruments, Winooski, VT, USA).

##### Luteinizing hormone (LH)

Serum LH was measured using Human LH ELISA kit (DRG International, Inc. USA, Catalog No. EIA-1289). The absorbance of each well was determined at 450 nm using (BioTek ELx800, BioTek Instruments, Winooski, VT, USA) within 30 min after adding the stop solution.

##### Total testosterone

Serum total testosterone concentration was measured using ELISA quantitative colorimetric method^107^, using a kit supplied by DiaMetra (Cat. No. DKO002, Perugia, Italy). The reaction was stopped by adding 100µL of stop solution to each well. The optical density (O.D.) was measured at λmax 450 nm against blank using (BioTek ELx800, BioTek Instruments, Winooski, VT, USA).

#### Semen analysis

Semen analysis was performed according to the World Health Organization (WHO) guidelines^104^. The following parameters were assessed: Macroscopic examination: Liquefaction time (normal: <60 min at room temperature); semen volume (mL) measured using a graduated pipette; pH measured using pH indicator paper; viscosity assessed by gentle aspiration with a wide-bore pipette. Microscopic examination: Sperm concentration was determined using a Neubauer hemocytometer. Samples were diluted 1:20 with fixative solution (0.6 M NaHCO₃, 0.4% (v/v) formaldehyde in distilled water). Counts were performed in duplicate, and results expressed as million/mL. Total sperm count was calculated as concentration × volume (million/ejaculate). Motility assessment, at least 200 spermatozoa were counted in duplicate and classified as: rapidly progressive (moving actively, either linearly or in a large circle, covering ≥ 25 μm/s); slowly progressive (moving actively but covering < 25 μm/s); non-progressive (motile in place, moving but not progressing); and immotile (no movement). Results were expressed as a percentage of each category. Sperm morphology, smears were prepared, air-dried, fixed, and stained using Diff-Quik staining kit (Catalog No. MOT-20-2, Medion Diagnostics, Switzerland). At least 200 spermatozoa were evaluated under oil immersion (1000× magnification) according to strict Kruger criteria^108^. Results were expressed as percentage of normal forms. OCTAX EyeWare™ version 2.2.2.318 was used for imaging and recording analysis.

#### Sperm apoptosis assessment by flow cytometry

Sperm apoptosis was evaluated using the Annexin V-FITC Apoptosis Detection Kit detection kit’s procedure (Beckman Coulter, Brea, CA, USA city, state abbreviation for USA, nation) and our standard protocol^109–111^. Semen samples were first subjected to density-gradient centrifugation (45%/100%) followed by washing and swim-up preparation. This procedure was specifically employed to enrich for motile spermatozoa and to remove the majority of leukocytes, round cells, somatic cells, cellular debris, and other contaminants before downstream molecular analyses.Spermatozoa washed twice with phosphate-buffered saline (PBS) and resuspended in 1× binding buffer at a concentration of 1 × 10⁶ cells/mL. 100 µL of cell suspension was transferred to a 5 mL flow cytometry tube, and 5 µL of Annexin V-FITC and 5 µL of PI were added. Tubes were gently vortexed and incubated for 15 min at room temperature in the dark. 400 µL of 1× binding buffer was added to each tube, and analysis was performed within 1 h using Beckman Coulter Epics XL flow cytometer equipped with 488 nm argon laser.

#### Sperm DNA fragmentation analysis

Sperm DNA fragmentation was assessed using colorimetric diphenylamine test to quantify DNA fragmentation in sperm cells according to our standard protocol^111–114^. Semen samples were first subjected to density-gradient centrifugation (45%/100%) followed by washing and swim-up preparation. This procedure was specifically employed to enrich for motile spermatozoa and to remove the majority of leukocytes, round cells, somatic cells, cellular debris, and other contaminants before downstream molecular analyses. For lysis, the cells were resuspended in 0.8 ml of 0.01 M PBS (pH 7.4), 0.7 ml of ice-cold lysis buffer (pH 8.0), 0.5% Triton X-100, 20 mM EDTA, and 5 mM Tris. To ensure complete lysis, the mixture was incubated at 4 °C for 15 min. The cell lysate was centrifuged at 4 °C and 13,000×g. The pellets contain complete DNA, whereas the supernatant now contains DNA fragments. The mixture was allowed to remain at room temperature for ten minutes after the supernatant was transferred into a 5-ml glass tube and 1.5 ml of 10% trichloroacetic acid (TCA) was added. Centrifugation at 500x g for 15 min at 4 °C was used to recover both intact and fragmented DNA. The inorganic phosphate was then released by boiling at 100 °C. Once more, the DNA pellet was suspended in 0.7 milliliters of 5% TCA and allowed to cool to room temperature. 0.5 ml of the supernatant was transferred to a new glass tube and incubated at 30 °C for the whole night after the suspensions were centrifuged at 300 × g for 4 °C. At a final concentration of 16 mg/mL, 1.5 g of diphenylamine was combined with 100 ml of acetic acid and 1.5 ml of H_2_SO_4_ with acetaldehyde. Colorimetric measurements were made at 600 nm to determine the absorbance intensity of the sample and the supernatant. DNA fragmentation^114^ was represented by the relative ratio of low-molecular-weight fragmented DNA to the overall DNA content of the sample.

#### Gene expression analysis by quantitative real-time PCR (qRT-PCR)

Total RNA was extracted from sperm pellets using TRIzol^®^ Reagent (Catalog No. 15596026, Invitrogen, Carlsbad, CA, USA) according to our previous protocol^115^. Semen samples were first subjected to density-gradient centrifugation (45%/100%), followed by washing and swim-up preparation. This procedure was specifically employed to enrich for motile spermatozoa and to remove the majority of leukocytes, round cells, somatic cells, cellular debris, and other contaminants before downstream molecular analyses. The resulting sperm pellet was then used for RNA extraction. RNA concentration and purity were assessed using NanoDrop 2000 spectrophotometer (Thermo Scientific, Wilmington, DE, USA). Samples with A260/A280 ratio between 1.8 and 2.0 were used for downstream applications. Complementary DNA (cDNA) was synthesized from 1 µg total RNA using High-Capacity cDNA Reverse Transcription Kit (Catalog No. 4368814, Applied Biosystems, Foster City, CA, USA) according to our previous protocol^115^. The genes were amplified using the HERAPLUS SYBR^®^ Green qPCR kit (Willowfort, Nottingham, UK). The 2^−ΔΔCT^ technique^116^ was used to analyze and quantify gene expression using Rotor-Gene Q (Qiagen, USA). Quantitative RT-PCR data were normalized using GAPDH as the endogenous control. This gene was selected based on its widespread use in previous human spermatozoa gene-expression studies^117–119^ and its consistent amplification performance across our samples. The stability of this reference gene was assessed by comparing Ct values across all study groups, and no significant variation was detected, supporting its use as an internal control for relative quantification. Table S1 lists the primer sequences that were utilized. The Bax/Bcl-2 ratio can be used as a prognostic indicator to determine whether a cell will experience apoptosis.

#### Protein expression analysis by ELISA

Protein expression levels of human caspase-3 (Cat. No. KHO1091, Thermofisher Scientific, USA), human cytochrome-c (Cat. No. BMS263, Thermofisher Scientific, USA), human Bax (Cat. No. EEL030, Thermofisher Scientific, USA), and human Bcl-2 (Cat. No. BMS244-3, Thermofisher Scientific, USA) were quantified in sperm lysates using commercially available ELISA kits. Semen samples were first subjected to density-gradient centrifugation (45%/100%) followed by washing and swim-up preparation. This procedure was specifically employed to enrich for motile spermatozoa and to remove the majority of leukocytes, round cells, somatic cells, cellular debris, and other contaminants before downstream molecular analyses. The resulting sperm pellet was then used for protein extraction. Lysates were centrifuged at 14,000 × g for 15 min at 4 °C, and supernatants were collected. Total protein concentration was determined using the BCA Protein Assay Kit (Catalog No. 23225, Thermo Scientific) to normalize sample loading. The absorbance was read at 450 nm within 15 min using a microplate reader (BioTek ELx800). Protein concentrations were calculated from standard curves generated using four-parameter logistic (4PL) curve fitting. Results were expressed as ng/mg total protein to normalize for loading differences. The Bax/Bcl-2 ratio was calculated by dividing Bax concentration by Bcl-2 concentration.

### Statistical analysis

Statistical analyses were performed using GraphPad Prism version 9.0 (GraphPad Software, San Diego, CA, USA). Shapiro-Wilks test and histograms will be used to evaluate the normality of the distribution of data. Quantitative parametric variables will be presented as mean and standard error of mean (SEM) and compared between the two groups utilizing unpaired Student’s t- test and compared between the four groups utilizing one- way ANOVA-test with post hoc test (Tukey). Quantitative non-parametric data will be compared between the two groups utilizing the Mann Whitney-test and compared between the four groups utilizing the Kruskal-Wallis test with the Mann Whitney-test to compare each group. All experiments were performed in replicates to ensure reproducibility. A two-tailed *P* < 0.05 will be considered statistically significant.

## Supplementary Information

Below is the link to the electronic supplementary material.

Supplementary Material 1

Supplementary Material 2

Supplementary Material 3

Supplementary Material 4

Supplementary Material 5

Supplementary Material 6

Supplementary Material 7

Supplementary Material 8

## Data Availability

All data generated or analyzed during this study are included in this published article [and its supplementary information files]. Any further datasets generated during and/or analyzed during the current study are available from the corresponding author on reasonable request.
