# Supplementary figures and images for: The link between diabetes and male infertility: mechanisms and implications
Source: Sci Rep. 2026 Jul 24;16:23198. doi: 10.1038/s41598-026-63095-w (PMC13400617; doi:10.1038/s41598-026-63095-w)

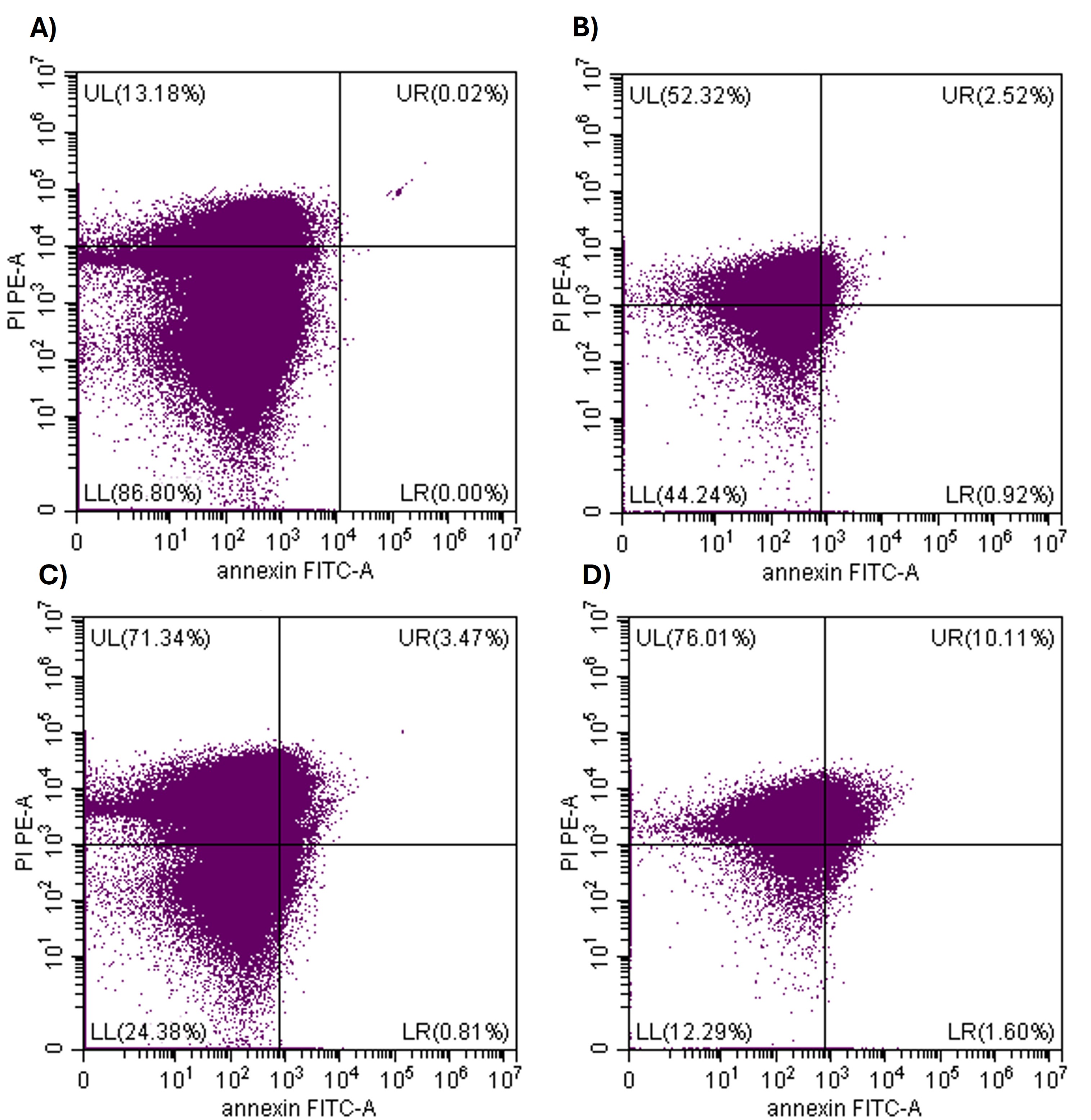

Supplement: Supplementary file 1 — Supplementary Material 1 [file 41598_2026_63095_MOESM1_ESM.jpg]
